# Annexin A1-derived peptide Ac_2-26_ in a pilocarpine-induced status epilepticus model: anti-inflammatory and neuroprotective effects

**DOI:** 10.1186/s12974-019-1414-7

**Published:** 2019-02-12

**Authors:** Alexandre D. Gimenes, Bruna F. D. Andrade, José Victor P. Pinotti, Sonia M. Oliani, Orfa Y. Galvis-Alonso, Cristiane D. Gil

**Affiliations:** 1 Department of Morphology and Genetics, Federal University of São Paulo (UNIFESP), São Paulo, SP 04023-900 Brazil; 2 Department of Molecular Biology, São José do Rio Preto School of Medicine (FAMERP), São José do Rio Preto, SP 15090-000 Brazil; 3 From the Post-Graduation in Biosciences, Instituto de Biociências, Letras e Ciências Exatas, São Paulo State University (IBILCE/UNESP), São José do Rio Preto, SP 15054-000 Brazil

**Keywords:** Cytokines, ERK, Fpr2, Glia, Hippocampus, Neuroinflammation

## Abstract

**Background:**

The inflammatory process has been described as a crucial mechanism in the pathophysiology of temporal lobe epilepsy. The anti-inflammatory protein annexin A1 (ANXA1) represents an interesting target in the regulation of neuroinflammation through the inhibition of leukocyte transmigration and the release of proinflammatory mediators. In this study, the role of the ANXA1-derived peptide Ac_2-26_ in an experimental model of status epilepticus (SE) was evaluated.

**Methods:**

Male Wistar rats were divided into Naive, Sham, SE and SE+Ac_2-26_ groups, and SE was induced by intrahippocampal injection of pilocarpine. In Sham animals, saline was applied into the hippocampus, and Naive rats were only handled. Three doses of Ac_2-26_ (1 mg/kg) were administered intraperitoneally (i.p.) after 2, 8 and 14 h of SE induction. Finally, 24 h after the experiment-onset, rats were euthanized for analyses of neuronal lesion and inflammation.

**Results:**

Pilocarpine induced generalised SE in all animals, causing neuronal damage, and systemic treatment with Ac_2-26_ decreased neuronal degeneration and albumin levels in the hippocampus. Also, both SE groups showed an intense influx of microglia, which was corroborated by high levels of ionised calcium binding adaptor molecule 1(Iba-1) and monocyte chemoattractant protein-1 (MCP-1) in the hippocampus. Ac_2-26_ reduced the astrocyte marker (glial fibrillary acidic protein; GFAP) levels, as well as interleukin-1β (IL-1β), interleukin-6 (IL-6) and growth-regulated alpha protein (GRO/KC). These effects of the peptide were associated with the modulation of the levels of formyl peptide receptor 2, a G-protein-coupled receptor that binds to Ac_2-26_, and the phosphorylated extracellular signal-regulated kinase (ERK) in the hippocampal neurons.

**Conclusions:**

The data suggest a neuroprotective effect of Ac_2-26_ in the epileptogenic processes through downregulation of inflammatory mediators and neuronal loss.

## Background

Epilepsy is a brain disease characterised by an enduring predisposition to generate epileptic seizures and by the neurobiological, cognitive, psychological and social consequences of this condition [1]. Temporal lobe epilepsy (TLE) is a type of focal epilepsy that has a great clinical relevance due to its high incidence and severity, and the commonest pathology underlying the TLE is unilateral hippocampal sclerosis associated with neuronal loss and gliosis [2]. These characteristics can be reproduced in animals using pilocarpine, a muscarinic receptor agonist [3]. In this model, the systemic or intracerebral application of pilocarpine induces the following steps: (1) an acute period that progressively develops in 24-h limbic status epilepticus (SE); (2) a silent period with progressive normalisation of behaviour and electroencephalogram, which varies from 4 to 44 days, and (3) a chronic period with recurrent spontaneous seizures [4, 5]. In addition, neuronal death and gliosis occur in the hippocampus and extrahippocampal regions, and the subsequent development of recurrent spontaneous seizures is similar to the development observed in complex partial seizures in humans [6–9].

Clinical and experimental evidence support the hypothesis that the inflammatory process in the brain is a common and crucial mechanism of epileptic seizures and epilepsy [10, 11]. The first evidence of the role of inflammation in human epilepsy was obtained clinically, showing that steroids and other anti-inflammatory drugs have anticonvulsant activity in patients refractory to conventional therapy [12]. Furthermore, increased serum levels of interleukins interleukin-1β (IL-1β), interleukin-6 (IL-6) and IL-1 receptor antagonists were detected in patients with extra-TLE and high levels of IL-1β in the TLE group, supporting the existence of a chronic inflammatory state in epileptic patients [13]. In nervous tissue, astrocytes and microglia are important sources of proinflammatory cytokines, such as IL-1β, IL-6 and TNF-α, and contribute to the epileptogenic process [10, 11]. However, the molecular mechanisms by which inflammation can increase the excitability of neurons are still unclear and open new perspectives for the treatment or prevention of these neurological diseases.

This scenario highlights annexin A1 (ANXA1), a 37 kDa protein that mimics the action of glucocorticoids by inhibiting the synthesis of eicosanoids and phospholipase A_2_, the leukocyte migration and the release of proinflammatory cytokines, thus contributing to the control of the inflammatory response [14]. In addition, increased levels of ANXA1 in the human brain, as well as in the activated glia (microglia and astrocytes) or scar tissue, have been described in different neurological pathologies, suggesting a role of this protein in response to neural injury [15]. Similarly, kainic acid-lesioned rat cerebellum presented increased levels of ANXA1 in the activated microglia at 24 h and later in the astrocytes (5 days) [16]. The neuroprotective role of ANXA1 was also demonstrated in a rat stroke model where administering the ANXA1 mimetic peptide (Ac_2-26_) decreased the size of the lesion and limited neutrophil infiltration [17, 18]. In addition, administering the recombinant human ANXA1 also could attenuate beta-amyloid-induced blood-brain barrier (BBB) impairment in vitro, suppressing microglial activation and clearing apoptotic neurons [19].

The biological actions of ANXA1 and its derived peptides can occur through functional interaction with formyl peptide receptors (Fpr), a family of G-protein-coupled receptors, and especially formyl peptide receptor 2 (Fpr2) [20, 21]. After binding to their agonists, these receptors activate a variety of signalling pathways, including intracellular calcium influx and activation of mitogen-activated protein kinases (MAPKs) which are important regulators of synaptic excitability and cognitive impairment in epilepsy [22]. These data reveal ANXA1 plays a significant role in the central nervous system diseases which, although of varying and often indefinite aetiology, share a common neuroinflammatory component. Thus, this study evaluates the role of pharmacological treatment with ANXA1-derived peptide Ac_2-26_ in the pilocarpine-induced status epilepticus (SE) in rats.

## Methods

### Animals

Adult male Wistar rats (200–250 g) were housed in a 12-h light-dark cycle with a controlled temperature (22 ± 2 °C) and relative humidity air between 40% and 60% and were allowed food and water ad libitum. Furthermore, the animals were carefully handled for 7 days prior to the initiation of the experiments for stress reduction. All procedures were approved by the Ethics Committee in Animal Experimentation of the Federal University of São Paulo - UNIFESP (CEUA No. 295805081) and agreed with the guidelines established by the National Council for the Control of Animal Experimentation (CONCEA).

### Induction of SE and pharmacological treatments

Rats were distributed into the following four groups: Naive (*n* = 12), Sham (*n* = 12), SE (*n* = 14) and SE+Ac_2-26_ (treated with the mimetic peptide of ANXA1; *n* = 12). Stereotaxic surgery was performed in animals from the Sham, SE and SE+Ac_2-26_ groups. They were then anaesthetized with acepromazine-ketamine-xylazine (1 mg/kg subcutaneously and 50 and 10 mg/kg intramuscularly, respectively) and received 1 ml/kg of veterinary pentabiotic (Fort Dodge, Campinas, SP, Brazil) to avoid infection. Cannula was implanted in the right posterior dorsal hippocampus with the following stereotaxic coordinates: AP − 5.9 mm, ML − 4.3 mm, and DV 3.5 mm [20].

Seven days after surgery, SE was induced according to previous studies [23, 24]. SE and SE+Ac_2-26_ groups received an intrahippocampal injection of pilocarpine (0.9 mg/animal; Sigma-Aldrich Corporation, St. Louis, MO, USA, Cat No. P6503-10G) diluted in 1 μl of sterile saline, while Sham received only 0.9% saline (1 μl). Also, ANXA1-derived peptide Ac_2-26_ (Ac-AMVSEFLKQAWFIENEEQEYVQTVK; Invitrogen, São Paulo, Brazil) was diluted in sterile saline and administered at 1 mg/kg intraperitoneally (i.p.) [25, 26], after 2, 8 and 14 h of SE induction. Doses of Ac_2-26_ were scaled up from the pilot study. In parallel, Sham and SE rats received 0.9% saline i.p., while Naive animals were only handled.

Each animal was placed in an individual acrylic box for behavioural assessment according to the Racine’s scale [27], for a period of 4 h after the onset of SE. SE was defined as continuous stage three or greater seizures and, for each rat, the SE type was labelled considering the predominant seizure type displayed for at least 2 h.

All animals received diazepam (DZP, 10 mg/kg; i.p.) 4 h after SE establishment. Naive and Sham groups were also injected with DZP in the same conditions, and animals submitted to SE were kept hydrated by subcutaneous injection of saline every 3 h. Then, 24 h after the pilocarpine injection, animals were euthanized by overdosage of sodium thiopental and the brains were collected.

### Analysis of neuronal degeneration

The animals were perfused via a cannula into the left ventricle of the heart with 0.9% saline followed by 4% phosphate-buffered paraformaldehyde. After perfusion, the brains were removed and fixed for an additional 4 h, subsequently dehydrated in ethanol 50% to 100% and xylene, and then embedded in paraffin. Brain coronal sections of 8 μm were obtained in a Leica RM2155 microtome (Leica Microsystems, Nussloch, Germany) and subsequently stained with haematoxylin-eosin (H&E) or Fluoro-Jade C (FJC) [28] for quantification of normal and degenerating neurons, respectively.

### Analysis of microglia and astrocytes in the hippocampus

For the localization of microglia and astrocytes in the hippocampus, immunohistochemistry was performed. Also, after an antigen retrieval step using citrate buffer (pH 6.0) at 96 °C for 30 min, endogenous peroxide activity was blocked, and the hippocampal sections were incubated overnight (~ 16 h) at 4 °C with the rabbit polyclonal antibody anti-Iba1 or anti-glial fibrillary acidic protein (GFAP; Novus Biological, Littleton, CO, USA, Cat No. NBP2-16908 and NB300-141), which are microglia and astrocyte markers, respectively, and are 1:1000 diluted in 2% BSA. After washing, the sections were incubated with a secondary biotinylated antibody (LAB-SA Detection kit, Invitrogen, Paisley, UK, Cat No. 95-9843). Positive staining was detected using a peroxidase-conjugated streptavidin complex, and colour was developed using 3,3′-Diaminobenzidine (DAB) substrate (Dako, Cambridge, UK, Cat No. K3468). Lastly, the sections were counterstained with haematoxylin.

### Cell counting

The quantifications of normal and degenerating neurons (FJC^+^ cells), as well as microglia and astrocytes, were performed in a blinded fashion using photomicrographs obtained in a × 40 objective on an Olympus microscope (Olympus Corporation, Tokyo, Japan). Cell density was then obtained according to Abercrombie’s method [29]. The quantification of cells was then performed in the right and left hippocampus to verify whether cannula implantation (right side) per se alters cell counting. For each animal, the anterior and posterior Cornu Ammonis (CA) 1, 3 and 4 and dentate gyrus were analysed using 5 quadrants of 50 × 50 μm, i.e. approximately 2500 μm^2^. The area was then determined using ImageJ software (National Institutes of Health, Bethesda, MD, USA), and the values were demonstrated as the mean ± standard error of the mean (SEM) of the number of cells per squared millimetre.

### Expression of Fpr2 and extracellular signal-regulated kinase (ERK) in the hippocampus

The analyses of Fpr2 and ERK expression in the hippocampus were performed using immunohistochemistry [26]. After an antigen retrieval step using citrate buffer (pH 6.0) at 96 °C for 30 min, endogenous peroxide activity was blocked, and the hippocampal sections were incubated overnight at 4 °C with the primary rabbit polyclonal antibody anti-Fpr2 (1:2000; Santa Cruz Biotechnology, CA, USA, Cat No. sc-57141) and mouse monoclonal anti-phosphorylated (p)ERK ½ (1:1000, Cell Signaling, Danvers, MA, EUA, Cat No. mAb #4370) diluted in 1% BSA. After washing, the sections were incubated with a secondary biotinylated antibody (LAB-SA Detection kit, Invitrogen, Paisley, UK, Cat No. 95-9843). Positive staining was then detected using a peroxidase-conjugated streptavidin complex, and colour was developed using a DAB substrate (Dako, Cambridge, UK, Cat No. K3468). Afterwards, the sections were counterstained with haematoxylin. Densitometric analyses for the Fpr2 immunostaining were then performed in the hippocampal neurons (n ≈ 5 animals/group), and 20 points were analysed in CA fields for an average related to the intensity of immunoreactivity [25, 26]. The values were subsequently obtained as arbitrary units (a.u.) between 0 and 255 using AxioVision software on an Axioskop 2-Mot Plus Zeiss microscope (Carls Zeiss, Jena, Germany), and the data were expressed as the mean ± SEM of arbitrary units.

### Analysis of cytokine and chemokine levels

Hippocampal samples were sonicated in a 50 mM Tris-HCl, 150 mM NaCl and 1% Triton-X pH 7.4 buffer containing a complete protease inhibitor cocktail and PhosSTOP tablets (Roche Applied Science, Mannheim, Germany, Cat No. 04906837001). Subsequently, samples were centrifuged at 10,000 × *g* for 20 min at 4 °C to obtain organ homogenates. For multiplex analysis, 25 μl of the hippocampal homogenates were employed using the MILLIPLEX MAP rat cytokine/chemokine panel (MILLIPLEX MAP RECYTMAG-65 K, Millipore Corporation, EUA, Cat No. #RECYMAG65K27PMX) and MAGPIX® Multiplexing Instrument (Millipore) according to the manufacturer’s instructions. Five analytes were studied in this work: IL-1β, IL-6, TNF-α (tumour necrosis factor-α), GRO/KC (growth-regulated alpha protein; also known as CXCL1) and MCP-1 (monocyte chemoattractant protein-1). The concentration of analytes was determined by MAGPIX Xponent software (Millipore Corporation, Billerica, MA, USA), and the results are reported as the mean ± SEM.

### Western blotting analysis

Protein levels of hippocampal homogenates were determined by Bradford assay and normalised prior to boiling in the Laemmli buffer (Bio-Rad Laboratories, USA, Cat No. #1610737). Pooled protein extracts (30 μg per lane) of hippocampus (*n* = 3 animals per group) from the indicated experimental conditions were loaded onto a 12% sodium dodecyl sulphate-polyacrylamide gel for electrophoresis together with appropriate molecular weight markers (Bio-Rad Life Science, USA, Cat No. 4110182) and transferred to ECL Hybond nitrocellulose membranes. Also, reversible protein staining of the membranes with 0.1% Ponceau-S in 5% acetic acid (Santa Cruz Biotechnology, CA, USA, Cat No. CAS 6226-79-5) was used to verify protein transfer. In this process, the membranes were incubated for 15 min in 5% BSA in Tris-buffered saline (TBS) prior to incubation with antibodies, and the primary antibodies used in this work were rabbit polyclonal anti-albumin (1:2000, Abcam, Cambridge, MA, USA, Cat No. ab10658), anti-Iba1 and anti-GFAP (1:500; Novus Biological, Littleton, CO, USA, Cat No. NBP2-16908 and NB300-141), anti-ANXA1 and anti-Fpr2 (1:200; Santa Cruz Biotechnology, CA, USA, Cat No. sc-12740 and sc-57141), anti-glyceraldehyde 3-phosphate dehydrogenase (GAPDH;1:5000; Sigma-Aldrich, St. Louis, Missouri, USA, Cat No. G9545-100UL), anti-ERK and mouse monoclonal anti-phosphorylated ERK1/2 (1:2000; Cell Signalling, Danvers, MA, EUA, Cat No. mAb #9102 and #4370), which all the antibodies were diluted in TBS with 0.1% Tween 20. For post-incubation with primary antibodies, membranes were washed for 15 min with TBS and subsequently incubated for 60 min at room temperature with the appropriate secondary antibodies. The secondary antibodies were peroxidase-conjugated rabbit anti-goat IgG, goat anti-rabbit (1:2000, Thermo Fisher Scientific Inc., MI, USA, Cat No. #31402 and #31460) or goat anti-mouse (1:2000, Millipore Corporation, CA USA, Cat No. 12-349). Finally, membranes were washed for 15 min with TBS, and immunoreactive proteins were detected (Westar Nova 2.0 chemiluminescent substrate kit; Cyanagen, Bologna, Italy, Cat No. XLS071,0250) using a GeneGnome5 chemiluminescence detection system (SynGene, Cambridge, UK). Proteins were then imaged and quantified using GeneTools software (SynGene) to determine the relative expression of indicated proteins (arbitrary units, a.u.).

### Statistical analysis

GraphPad software version 6.00 (GraphPad Software, La Jolla, CA, USA) was used for the statistical analysis, and normality was determined by performing the Kolmogorov-Smirnov test. In samples with a normal distribution, the analysis of variance (ANOVA) was applied and then the Bonferroni post-test was performed. In contrast, the Kruskal-Walls test followed by the Dunn test was used for samples with a non-normal distribution. In all cases, a *P* value < 0.05 was considered significant.

## Results

### Systemic treatment with Ac_2-26_ decreases loss of hippocampal neurons in the SE

Behavioural analysis showed that all rats of the SE groups, treated or not with Ac_2-26_ peptide, displayed seizures with Racine’s score 3 to 5 and were characterised as generalised SE (Table 1). Animals from Naive and Sham groups did not show any type of seizure. During and after SE, rats’ survival rate was 100% and, 24 h after pilocarpine application, locomotion and rats’ self-feeding was normal. After DZP administration, no seizures were detected in the rats from SE groups.

**Table 1:** Racine’s score during 4 h of SE induction

Groups		Racine’s score				
		1	2	3	4	5
Naive	*n* = 12	0	0	0	0	0
Sham	*n* = 12	0	0	0	0	0
SE	*n* = 14	0	0	6	1	7
SE+Ac_2-26_	*n* = 12	0	0	5	0	7

Rats were distributed into the following four groups: Naive (*n* = 12), Sham (*n* = 12), SE (*n* = 14) and SE+Ac_2-26_ (treated with the mimetic peptide of ANXA1; *n* = 12). Each animal was placed in an individual acrylic box for behavioural assessment for a period of 4 h after the onset of SE, and the following predominant seizure type was analysed according to the Racine’s scale [27]: 1—mouth and facial movement, 2—head nodding, 3—forelimb clonus, 4—rearing with forelimb clonus, 5—rearing and falling with forelimb clonus (generalised motor convulsions)

**Fig. 1:**
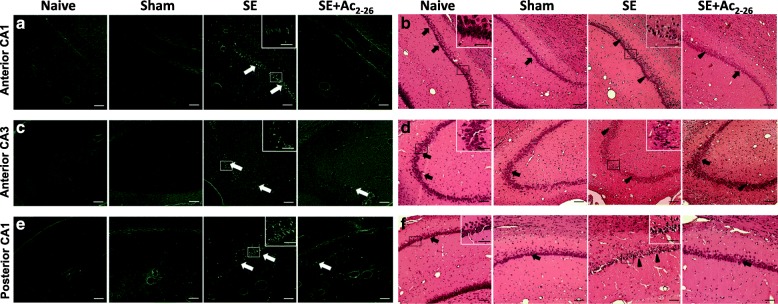
Ac_2-26_ decreases loss of hippocampal neurons in the SE model. **a**, **c**, **e** Representative photomicrographs of FJC staining show degenerating neurons (white arrows) in the anterior and posterior hippocampal CA1 and CA3 regions 24 h after of SE-onset. Few FJC^+^ cells (white arrows) were detected in the SE+Ac_2-26_ group. No FJC signal was observed in the hippocampus of control rats (Naive and Sham). **b**, **d**, **f** Healthy neurons (black arrows) and picnotic cells (black arrowheads), the latter were more evident in the SE group. Insets, details of degenerated and healthy neurons from squared areas. Stain, Fluoro Jade-C (**a**, **c**, **e**) and haematoxilin-eosin (**b**, **d**, **f**). Bars, 200 μm; 40 μm (insets)

In addition, neurodegenerative alterations in the hippocampus were characterised using FJC staining. At 24 h post-SE, significant neuronal injuries of pyramidal cells in anterior CA1, CA3 and posterior (dorsal and ventral) CA1 regions were evident (Fig. 1a, c, e). The systemic treatment with Ac_2-26_ was associated with very few degenerating neurons and no FJC^+^ cells were detected in the control groups (Naive and Sham) (Fig. 1a, c, e). H&E stained sections of the CA regions confirmed these findings, showing neurons with pyknotic nuclei in the SE group, while SE+Ac_2-26_ and control groups presented a predominance of cells with a normal aspect, euchromatic nucleus and evident nucleolus (Fig. 1b, d, f).

**Fig. 2:**
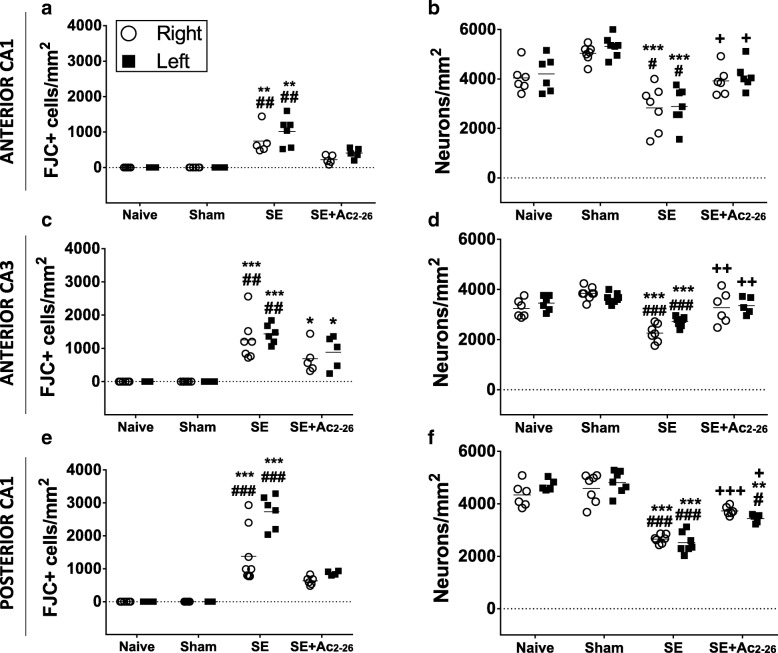
Quantification of hippocampal neurons in CA1 (anterior and posterior) and CA3 regions. Degenerated (FJC^+^cells) (**a**, **c**, **e**) and healthy neurons (**b**, **d**, **f**) were counted in the right and left sides of hippocampus. Dot plot graphs represent number of cells/mm^2^ (*n* = 8 animals/group). ^#^*P* < 0.05; ^##^*P* < 0.01 and ^###^*P* < 0.001 versus *Naive*. *P < 0.05; **P < 0.01 and ***P < 0.001 versus *Sham.* ^+^*P* < 0.05; ^++^*P* < 0.01 and ^+++^*P* < 0.01 versus SE (Kruskal-Wallis, Dunn post-test)

The quantification of degenerated neurons (FJC^+^ cells) and healthy neurons (H&E stain) were then performed in the right and left hippocampus to verify whether cannula implantation (right side) per se alters cell counting. As expected, SE produced a marked increase in the number of FJC^+^ cells in the anterior regions of CA1, CA3 and posterior regions of CA1 compared to the Naive and Sham groups (Fig. 2a, c, e). Also, pharmacological treatment with Ac_2-26_ resulted in a diminished number of FJC^+^ cells in the anterior CA1, CA3 and posterior CA1 regions in relation to the untreated SE group and presented no significant differences between control groups. These findings were corroborated by the higher number of healthy cells in the SE+Ac_2-26_ group compared to the untreated SE group (Fig. 2b, d, f), and the analysis of CA4/dentate gyrus regions showed similar aspects in the neurodegenerative alterations between SE and SE+Ac_2-26_ groups (data not shown).

### Ac_2-26_ does not reduce the hippocampal gliosis induced by SE

**Fig. 3:**
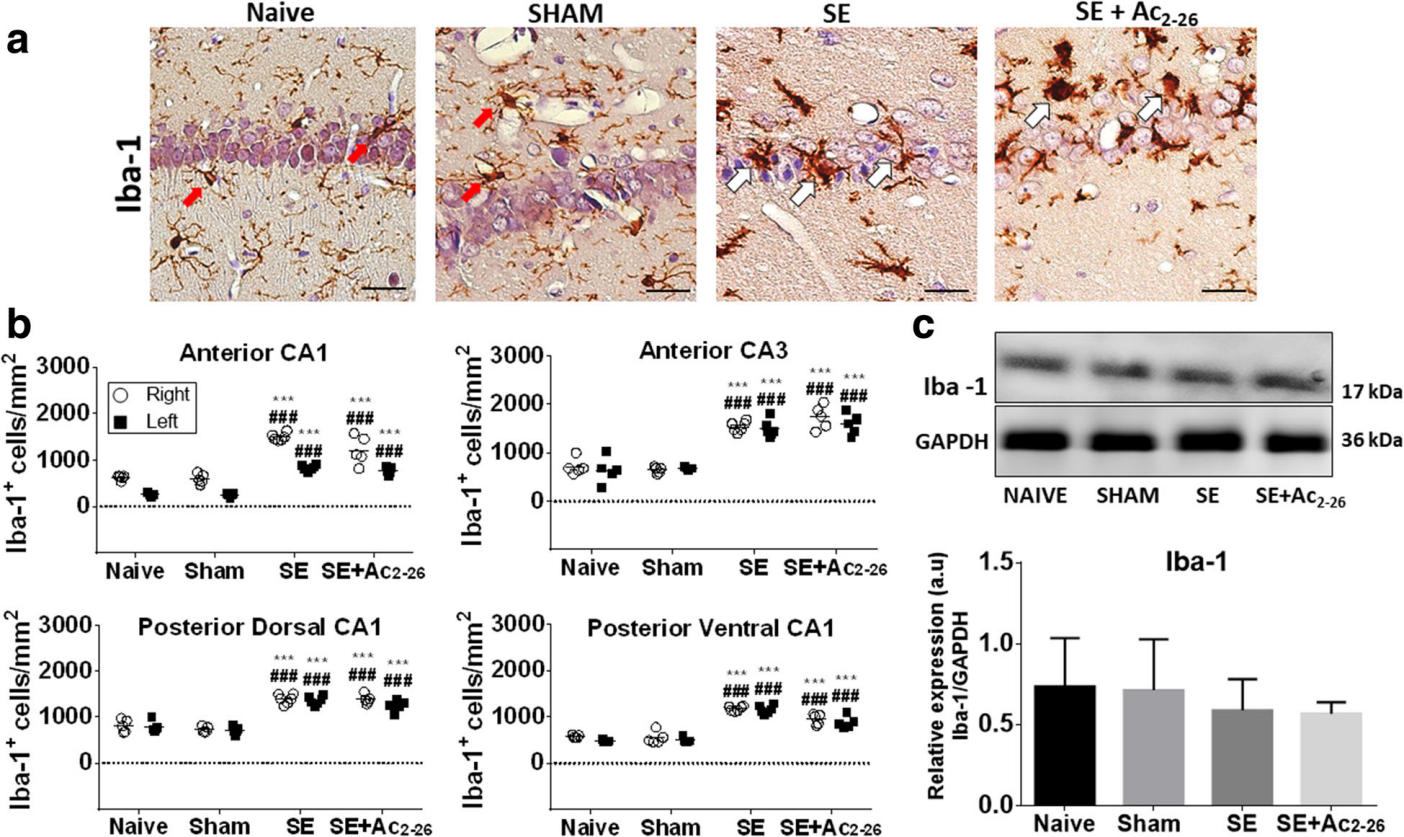
Ac_2-26_ does not reduce the microglia recruitment induced by SE. **a** Resting microglia from control groups (Naive and Sham) characterised by a fully ramified form (red arrows). SE and SE+Ac_2-26_ groups presented bushy and ameboid cells with few and short cytoplasmic prolongations (white arrows). Counterstain, Haematoxylin. Bars, 50 μm. **b** Quantification of Iba-1^+^cells in the in CA1 (anterior and posterior) and CA3 regions, right and left sides. Dot plot graph of Iba-1^+^ cells/mm^2^ (*n* = 6 animals/group). ^###^*P* < 0.001 vs. Naive; ***P < 0.001 vs. SHAM (Kruskal-Wallis, Dunn post-test). **c** Western blot analysis to measure Iba-1 (~ 17 kDa) in the pooled extracts of rat hippocampus (*n* = 3 animals/group) from all experimental groups. GAPDH was used as a protein loading control. Immunoreactive bands for Iba-1 were semi-quantified by densitometry and are expressed as arbitrary units (a.u.) of the ratio of Iba-1/GAPDH (data represent one illustrative blot from four independent experiments; mean ± SEM)

The microglia population of the control groups presented a fully ramified form that characterises resting cells (Fig. 3a) [30]. At 24 h post-SE with or without Ac_2-26_ treatment, microglia activation in the hippocampus was evidenced by the presence of bushy and ameboid cells with few and short cytoplasmic prolongations (Fig. 3a).

The results show that the number of microglia cells (Iba-1^+^ cells) increased in the anterior and posterior regions of CA1 and CA3 of SE and SE+Ac_2-26_ groups compared to that of the controls (Naive and SHAM; Fig. 3b). Additionally, microglia counts were similar in the right and left hippocampi. Despite a marked increase in the microglia number, no differences in the ionised calcium binding adaptor molecule 1 (Iba-1) levels of the hippocampal homogenates were demonstrated among the experimental groups (Fig. 3c).

**Fig. 4:**
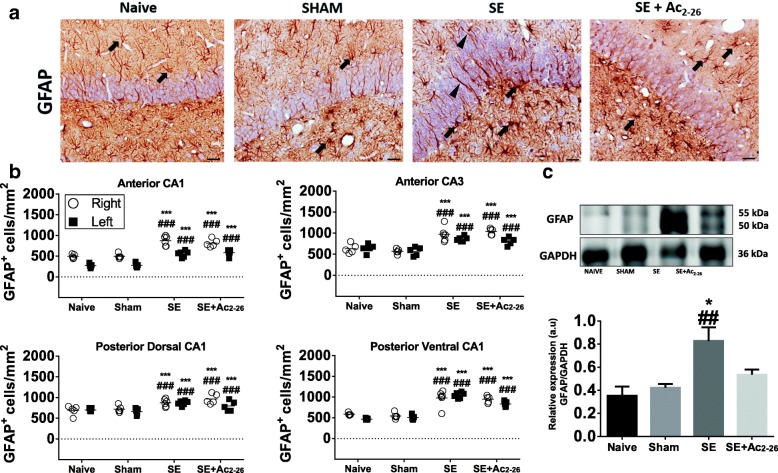
Astrocyte analysis in the pilocarpine-induced SE model. **a** GFAP^+^ cells (arrows) were detected in the hippocampus of all experimental groups. SE group presented profuse reactive astrogliosis (arrowheads) compared to the controls and SE+Ac_2-26_ groups. Counterstain, Haematoxylin. Bars, 50 μm. **b** Quantification of GFAP^+^ cells in the CA1 (anterior and posterior) and CA3 regions, right and left sides. Dot plots represent GFAP^+^ cells/mm^2^ (n = 6 animals/group). ^###^*P* < 0.001 vs. Naive; ***P < 0.001 vs. SHAM (Kruskal-Wallis, Dunn post-test). **c** Western blot analysis to measure GFAP (~ 50–55 kDa) in the pooled extracts of rat hippocampus (*n* = 3 animals/group) from all experimental groups. GAPDH was used as a protein loading control. Immunoreactive bands for GFAP were semi-quantified by densitometry and are expressed as arbitrary units (a.u.) of the ratio of GFAP/GAPDH (data represent one illustrative blot from four independent experiments). ^##^*P* < 0.01 vs. Naive; *P < 0.001 vs. Sham (ANOVA, Bonferroni post-test)

Furthermore, the results show profuse reactive astrogliosis in the anterior and posterior CA1 and CA3 regions, with increased GFAP levels being detected in the SE group compared to the controls (Fig. 4a–c). Despite similar findings regarding the quantifications of GFAP^+^ cells between SE and SE+Ac_2-26_ groups, the latter showed decreased levels of hippocampal GFAP (Fig. 4a–c). Also, cannula implantation per se did not create any difference between right and left sides of the hippocampus in relation to the cell counts of all experimental groups.

### Systemic treatment with Ac_2-26_ reduces the proinflammatory cytokine and GRO/KC levels in the hippocampus

**Fig. 5:**
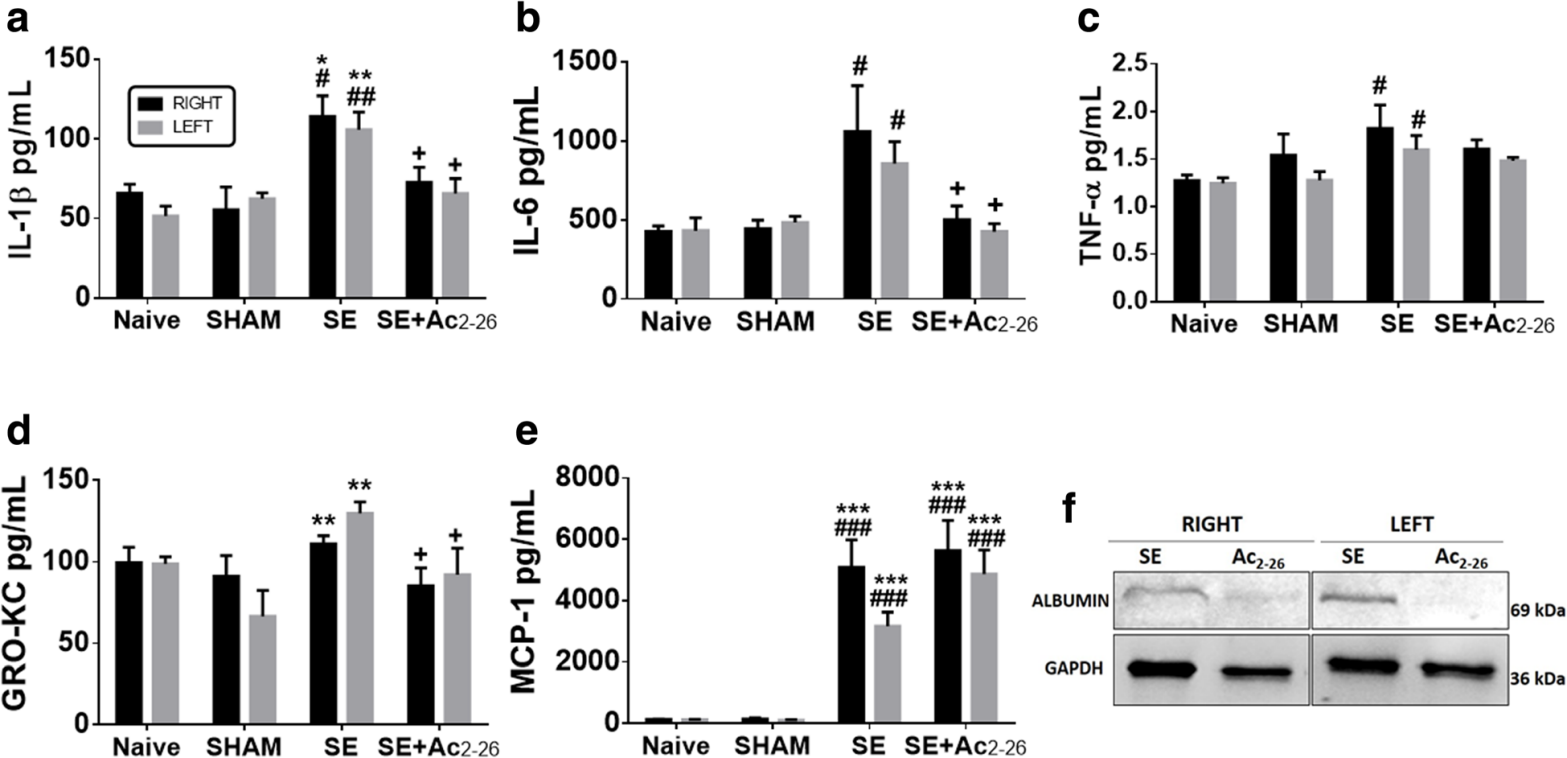
Effect of Ac_2-26_ in the cytokine and chemokine levels in the hippocampus. **a** IL-1β. **b** IL-6. **c** TNF-α. **d** GRO/KC. **e** MCP-1. Data represent the mean ± SEM of cytokine/chemokine dosage (pg/mL) (*n* = 5 animals/group). ^**#**^*P* < 0.05, ^**##**^*P* < 0.01 and ^**###**^*P* < 0.001 versus Naive; *P < 0.05, **P < 0.01 and ***P < 0.001 vs. Sham; ^**+**^*P* < 0.05 vs. SE (Kruskal-Wallis, Dunn post-test). **f:** Western blot analysis to measure albumin (~ 69 kDa) in the pooled extracts of rat hippocampus (*n* = 3 animals/group). GAPDH was used as a protein loading control. Immunoreactive bands for albumin were semi-quantified by densitometry and are expressed as arbitrary units (a.u.) of the ratio of albumin/GAPDH (data represent one illustrative blot from four independent experiments)

The analysis of the cytokines and chemokines revealed that SE increased levels of IL-1β, IL-6, TNF-α, GRO/KC and MCP-1 in the hippocampal homogenates in relation to the control groups, indicating local inflammatory response (Fig. 5a–e). In contrast, systemic treatment with Ac_2-26_ reduced IL-1β, IL-6 and GRO/KC levels in relation to untreated SE and presented a similar production of TNF-α of control groups (Fig. 5a–d). However, administration of Ac_2-26_ maintained high levels of hippocampal MCP-1, as detected for the untreated SE group (Fig. 5e).

In addition, immunoblot analysis showed increased levels of albumin in the right and left hippocampus of SE group compared to the SE+Ac_2-26_, suggesting a protective effect of peptide in the disruption of BBB (Fig. 5f).

### Ac_2-26_ decreased Fpr2 levels in hippocampal neurons and ERK activation

**Fig. 6:**
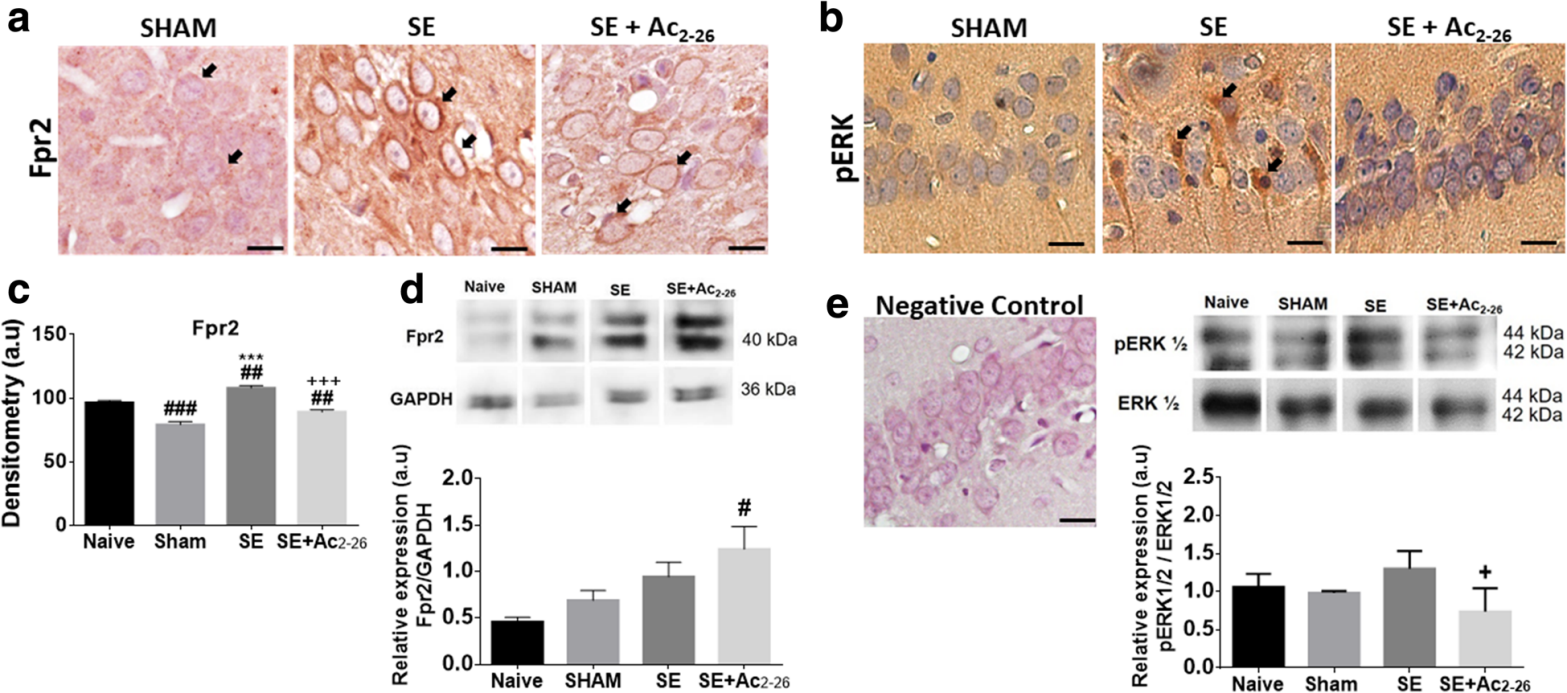
Ac_2-26_ decreased Fpr2 and ERK levels in hippocampal neurons. **a**, **b** SE group showed hippocampal neurons with intense immunostain for Fpr2 and phosphorylated ERK (arrows) compared to the SHAM and SE+Ac_2-26_ groups. No immunostaining was detected in the sample used as a negative control. Counterstain, haematoxylin. Bars, 20 μm. **c** Densitometric analysis of Fpr2 expression in the hippocampal neurons showed increased levels of this receptor, abrogated by Ac_2-26_ treatment. Data represent mean ± SEM of Fpr2 expression (a.u.; n = 5 animals/group). ^**##**^*P* < 0.01, ^**##**^*P* < 0.001 vs. Naive; ***P < 0.001 vs. Sham; ^**+++**^*P* < 0.05 vs. SE (Kruskal-Wallis, Dunn post-test). **d, e:** Immunoassays for Fpr2 (~ 40 kDa) and pERK (~ 42–44 kDa) detection in the pooled extracts of rat hippocampus (n = 3 animals/group) from all experimental groups. GAPDH and total ERK were used as protein loading controls. Immunoreactive bands for proteins were semi-quantified by densitometry and are expressed as arbitrary units (a.u.) of the ratio of Fpr2/GAPDH or pERK/ERK (data represent one illustrative blot from four independent experiments). ^#^*P* < 0.05 vs. Naive, ^+^*P* < 0.05 vs. SE (ANOVA, Bonferroni post-test)

The hippocampal neurons from the SE group exhibited intense immunostaining for Fpr2 in comparison to the control and SE+Ac_2-26_ groups (Fig. 6a). The densitometric analysis then confirmed the immunohistochemistry findings, showing a marked increase of Fpr2 in the SE condition, which this effect was decreased by the systemic treatment with Ac_2-26_ (Fig. 6c). However, no differences in the Fpr2 levels were detected in the hippocampal homogenates from the SE and SE+Ac_2-26_ groups (Fig. 6d).

In addition, strong immunoreactivity for phosphorylated ERK (pERK) was exhibited in the hippocampal neurons from the SE group in relation to the other groups (Fig. 6b). Also, no immunostaining was detected in the sample used as negative control (Fig. 6e). Lastly, the immunoblot analysis of hippocampal homogenates showed that systemic treatment with Ac_2-26_ decreased levels of the pERK in relation to the untreated SE group, confirming histological findings (Fig. 6f).

## Discussion

This study evaluated the effect of pharmacological treatment with anti-inflammatory ANXA1-derived peptide in a pilocarpine-induced SE model in rats. Using histological, histochemical, biochemical and molecular analyses, the results showed that systemic treatment with Ac_2-26_ reduced neuronal injury and inflammation related to SE.

As expected, at 24 h post-SE, rats that presented generalised convulsive SE (Racine’s score 3 to 5) showed the loss of bilateral hippocampal neurons, which was confirmed by reduced healthy neuron counting. Also, the bilateral lesion observed in the SE group corroborates previous data in which neurodegeneration and glial alterations occurred only ipsilaterally to the injection of pilocarpine in a generalised way since the neuronal circuitry interconnects several regions of the brain [24, 31]. Interestingly, systemic treatment of rats with Ac_2-26_ produced a neuroprotective effect in the areas of anterior and posterior (dorsal and ventral) CA1 and anterior CA3. These hippocampal regions are the main areas affected with neuronal loss that present the classic pattern of hippocampal sclerosis in patients with TLE [32–34], suggesting an important effect of the ANXA1-derived peptide in the SE model.

In addition to the neuronal loss, pilocarpine induced-SE produced a marked increase in the microglia and astrocyte counts in all analysed areas of the hippocampus, and this effect was not reversed by Ac_2-26_ treatment. These findings were corroborated by increased hippocampal levels of MCP-1 in the SE and SE+Ac_2-26_ groups. Then, MCP-1 released by astrocytes and endothelial cells participates in the recruitment of activated monocytes and lymphocytes in the central nervous system, acting as an important mediator in brain inflammation [35, 36]. Gliosis is a common feature of the brains of patients and animal models of seizures and epilepsy, and if this condition is not resolved in the post-acute or pre-chronic period, it has an inhibitory effect on nervous tissue regeneration after injury [37–39]. In this regard, hippocampal microglia from the SE and SE+Ac_2-26_ groups showed bushy and ameboid aspects with few and short cytoplasmic prolongations, suggesting its activation state [39]. Studies have shown that microglia releases ANXA1, in contrast to the astrocytes [16]. Additionally, Ac_2-26_ can induce the activation and migration of the microglia to solve the inflammation [40]. Furthermore, systemic pilocarpine-induced SE in rats increased ANXA1 levels in the brain in the acute phase (24 h), gradually decreasing in the latency period (72 h to 2 weeks) and then increasing in the chronic phase (30 days), suggesting a regulatory role of ANXA1 in epilepsy [41]. Together, these data indicate that the high levels of MCP-1 in the SE model cause microglia recruitment to the hippocampus, contributing to the release of ANXA1 and consequent regulation of epileptogenesis.

Despite the detection of astrogliosis in the SE and SE+Ac_2-26_ groups, levels of hippocampal GFAP were reduced after treating with Ac_2-26_. The discrepancy in the results can be explained by the methods of analysis adopted, especially for quantifying the cells, in which only the cell bodies were considered, decreasing the profusion of cytoplasmic prolongations, which this effect was more evident in the SE group. In fact, astrocytes in the inflamed brain undergo hypertrophy of cellular processes, attenuating their stellate morphology, and is associated with GFAP upregulation and the reactive state [42, 43]. Although reactive astrocytes can be beneficial in acute injuries and chronic neurological diseases through formation of scar that encapsulates injury, seals damaged BBB and provides trophic support to regenerating axons, other forms of astrocyte reactivity appear to be harmful [43, 44]. Some studies indicated astrocytes have an important role in the generation and spread of seizure activity [42, 45, 46]. For example, a recent study showed that astrocyte-derived amyloid-β (Aβ) peptides can mediate the degeneration of neurons through the activation of glutamatergic *N*-methyl-d-aspartate (NMDA) receptors in a model of TLE triggered by systemic administration of kainic acid [46]. In vitro, kainic acid reduces neuronal viability more in neuronal/astrocyte co-cultures than in pure neuronal culture, and this effect attenuated by precluding Aβ production [46]. Considering upregulation of GFAP is a classical hallmark of reactive astrogliosis, Ac_2-26_ may be involved in the regulation of SE-induced reactive astrocytes and neuronal degeneration in response to pilocarpine-induced SE in the rat hippocampus.

Consistent with these findings, Ac_2-26_ treatment also produced decreased levels of albumin in the rat hippocampus in relation to the untreated SE group, confirming the protective role of ANXA1 in the integrity of BBB [47, 48]. Increased albumin levels in the brain, a marker of BBB leakage, have been associated with the generation of seizures and epileptogenesis [49, 50]. Additionally, proinflammatory mediators can induce and sustain BBB disruption by affecting the endothelial integrity and can have a role in seizure activity by modifying the excitability and seizure thresholds [50, 51]. In this regard, the present study has shown that pilocarpine-induced SE increased the levels of pro-inflammatory and neurotoxic cytokines IL-1β, IL-6, TNF-α and chemokine GRO/KC in the rat hippocampus. In contrast, systemic treatment with Ac_2-26_ reduced the IL-1β, IL-6 and GRO/KC levels, confirming its anti-inflammatory role as having been demonstrated in other experimental models of neuroinflammation and uveitis and ocular allergies [17, 18, 25, 26, 52]. Inflammation plays a crucial role in the generation of epileptic seizures, as demonstrated in an animal model resistant to epileptogenesis, the neotropical rodent *Proechimys* [53]. After systemic pilocarpine-induced SE, neotropical rodents showed no changes in IL-1β, IL-6, IL-10, TNF-α and VEGF levels in the hippocampus and cortex compared to the control group. However, Wistar rats, which develop SE, presented a significant increase of these cytokines, except IL-10, in relation to the neotropical rodents.

The anti-inflammatory effect of the ANXA1-Fpr2 system was evidenced in a murine model of endotoxin-induced cerebral inflammation [54]. Also, ANXA1- or Fpr2/3-null mice present more exacerbated inflammatory responses induced by LPS, such as leukocyte adhesion to the endothelium and generation of proinflammatory mediators. These effects were abrogated by treatment with Ac_2-26_ in the ANXA1-null mice but not in Fpr2/3. In our study, Fpr2 expression was detected in the hippocampal neurons of all experimental groups, corroborating previous data [40]. In addition, after 24 h of SE, immunohistochemical studies showed a significant increase in the Fpr2 levels in the neurons in relation to the controls, and this effect was reverted by the treatment with the peptide Ac_2-26_. Diminished expression of neural Fpr2 after peptide administration is consistent, and once activated by the ligand, this receptor undergoes rapid phosphorylation and are desensitised and internalised [55]. Furthermore, the binding of different agonists (amyloid-β_1–42_ oligomer, fMLF or MMK1) and Fpr2 increased the generation of the reactive oxygen species (ROS) in the adult hippocampal neural stem/progenitor cells [56, 57]. The amyloid-β_1–42_ oligomer also triggered senescent phenotype of neuronal stem/precursor cells (NSPCs), as well as inhibited cell proliferation and differentiation [56]. Considering these findings, the ANXA1-Fpr2 system may be operative in the SE model as a tool to protect neurons against cell death.

On the other hand, western blot analyses revealed increased levels of Fpr2 in the hippocampal homogenates after peptide treatment. The discrepancy observed between the immunohistochemistry and western blot can be explained by the fact that the hippocampus presents other cell types that also express Fpr2, especially astrocytes and microglia [58].

The binding of specific agonists to Fprs triggers several intracellular signalling cascades, including the MAPK pathway, which have key roles in several biological functions, such as angiogenesis, cell proliferation and protection against cell death [55]. Then, ERK levels were investigated to better understand the molecular mechanisms involved in the ANXA1-derived peptide in the SE model. The results indicate that SE is associated with increased ERK levels in the hippocampal neurons while administration of Ac_2-26_ reduced ERK phosphorylation. ERK activation in epilepsy stimulates the expression of NMDA receptors, causing synaptic excitability and, in turn, leading to seizures [59].

## Conclusions

Altogether, the data support that ANXA1-derived peptide attenuates the increase of astrocyte activity and release of pro-inflammatory cytokines and mitigates the severity of brain damage in the SE model by regulating Fpr2/ERK signalling pathways. These results may be of significance for the explanation of epileptogenesis and provide valuable information about the ANXA1-Fpr2 system as an important therapeutic target for TLE.

## Abbreviations

Ac_2-26_: ANXA1 N-terminal-derived peptide
ANXA1: Annexin A1
BBB: Blood-brain barrier
CA: Cornu Ammonis
DAB: 3,3′-Diaminobenzidine
DZP: Diazepam
ERK: Extracellular signal-regulated kinase
FJC: Fluoro-Jade C
Fpr2: Formyl peptide receptor 2
GAPDH: Glyceraldehyde 3-phosphate dehydrogenase
GFAP: Glial fibrillary acidic protein
GRO/KC: growth-regulated alpha protein
H&E: Haematoxylin-eosin stain
Iba-1: Ionised calcium binding adaptor molecule 1
IL-1β: Interleukin-1β
IL-6: Interleukin-6
MCP-1: Monocyte chemoattractant protein-1
NMDA: Glutamatergic *N*-methyl-d-aspartate
pERK: Phosphorylated extracellular signal-regulated kinase
SE: Status epilepticus
TLE: Temporal lobe epilepsy
TNF-α: Tumour necrosis factor-α
